# Feature Selection via Swarm Intelligence for Determining Protein Essentiality

**DOI:** 10.3390/molecules23071569

**Published:** 2018-06-28

**Authors:** Ming Fang, Xiujuan Lei, Shi Cheng, Yuhui Shi, Fang-Xiang Wu

**Affiliations:** 1 School of Computer Science, Shaanxi Normal University, Xi’an 710119, China; mfang@snnu.edu.cn (M.F.), cheng@snnu.edu.cn (S.C.); 2 Department of Computer Science and Engineering, Southern University of Science and Technology, Shenzhen 518055, China; shiyh@sustc.edu.cn; 3 Department of Mechanical Engineering and Division of Biomedical Engineering, University of Saskatchewan, Saskatoon, SK S7N 5A9, Canada

**Keywords:** feature selection, essential protein, flower pollination algorithm, machine learning, protein-protein interaction (PPI) network

## Abstract

Protein essentiality is fundamental to comprehend the function and evolution of genes. The prediction of protein essentiality is pivotal in identifying disease genes and potential drug targets. Since the experimental methods need many investments in time and funds, it is of great value to predict protein essentiality with high accuracy using computational methods. In this study, we present a novel feature selection named Elite Search mechanism-based Flower Pollination Algorithm (ESFPA) to determine protein essentiality. Unlike other protein essentiality prediction methods, ESFPA uses an improved swarm intelligence–based algorithm for feature selection and selects optimal features for protein essentiality prediction. The first step is to collect numerous features with the highly predictive characteristics of essentiality. The second step is to develop a feature selection strategy based on a swarm intelligence algorithm to obtain the optimal feature subset. Furthermore, an elite search mechanism is adopted to further improve the quality of feature subset. Subsequently a hybrid classifier is applied to evaluate the essentiality for each protein. Finally, the experimental results show that our method is competitive to some well-known feature selection methods. The proposed method aims to provide a new perspective for protein essentiality determination.

## 1. Introduction

Essential proteins are required for the viability of an organism. The ability to predict essential proteins has the potential to influence the understanding of minimal requirements for a cell and the search for drug targets [1]. The proteins are regarded as essential if their deletion leads to lethality, while nonessential proteins are those whose knockouts do not kill the organisms [2].

So far, the prediction of protein essentiality has been supported by a large number of experimental methods like gene knockouts [3], RNA interference [4], and conditional knockouts [5]. However, the execution of each of these methods needs a large amount of time and funds. Taking these constraints into account, more and more researches on the identification of protein essentiality have been carried out using computational methods, especially with the growth and development of available data. 

The previous studies found that the essentiality of a protein had crucial relationships with its protein connectivity in the PPI network, which is viewed as topological features, as well as its biological features. From a topological perspective, a study revealed correlation between degree centrality (DC) in the PPI networks and essentiality [6]. Similarly, Betweenness Centrality (BC) [7], Closeness Centrality (CC) [8], Eigenvector Centrality (EC) [9], Information Centrality (IC) [10], and Subgraph Centrality (SC) [11] were also found to be closely related to the essentiality. However, these methods had their limitations in that they consider only topological features. With the emergence of more biology data, these data were integrated with the topology information to predict the essentiality of proteins from a biological perspective. For example, researchers suggested that the essentiality was closely tied to orthology [12], and the CoEWC method was proposed by combining gene expression and the PPI network, which considers the clustering properties of the neighbors of a protein [13]. A recent study used protein complex to predict protein essentiality based on the ideas that the proteins in protein complexes tend to be essential compared to those not contained in any complexes, and the proteins in multiple complexes are more likely to be essential compared with those that only exist in an individual complex [14]. Most of these methods predicted protein essentiality by attaching a score to each protein, and then the proteins with higher scores were identified as the essential proteins. The identification of protein essentiality was also implemented using machine learning methods. For example, the Naïve Bayes classifiers were utilized by using some features containing topological and biological features [15]. A study examined the relationship between sequence characteristics and the gene essentiality and found that there was a strong interrelation between them. A Support Vector Machine (SVM) classifier was constructed to predict essential genes in two model organisms based on the network topological features, such as DC, BC, CC, and clustering coefficient, and the sequence properties, such as open reading frame (ORF) length, strand, and phyletic retention [16]. A decision tree–based meta-classifier that integrated eight decision tree classifiers was constructed to predict essential genes based on network topology, subcellular localization, and biological process information [17]. The SVM-RFE was used to select some features including topological features, composited features, and subcellular localization information to identify essential proteins [18].

Feature selection is an important measure to reduce the negative impact of superfluous features. The aim of feature selection is to select the optimal subset by deleting those redundant features, which is a crucial step toward performing machine learning. Search strategies of feature selection include exhaustive search, heuristic search, and random search. In an exhaustive search, all the possible features should be estimated so as to decide the best subset of features, it means that the 2*^k^* possible subsets of features are assessed where *k* represents the number of original features [19]. Unfortunately, evaluating all the features would result in huge cost and time consumption. The heuristic search, such as sequential forward selection (SFS) [20] and sequential backward selection (SBS) [21], can find a balance between search performance and computation efficiency to reduce the time consumption of an exhaustive search. SFS begins with empty set, and the features are added to the subset sequentially until the set meets the requirement, while SBS begins with full set, and the features are deleted from the subset sequentially until the set meets the requirement. The emergence of the random search can improve the efficiency of feature selection to some extent. mRMR was proposed for feature selection [22]. The MRMD feature extraction method balanced accuracy and stability of feature ranking and prediction task which can improve the efficiency of classification [23]. A recent paper used MRMD to reduce the features dimension and obtained better performance [24]. Moreover, feature selection problems can be solved through swarm intelligence algorithms because its substance is an optimization problem. There are many different versions of swarm intelligence algorithms for feature selection. The particle swarm optimization (PSO) algorithm was proposed in 1995 [25], and then it was widely used for feature selection problems [26,27,28]. In addition, the genetic algorithm (GA) [29,30,31], ant colony optimization (ACO) [32,33], and firefly algorithm (FA) [34] were also used to address the problem of feature selection. The recently proposed flower pollination algorithm (FPA) was extended to apply for feature selection [35].

The FPA algorithm has previously been used for the identification of essential proteins [36]. We herein intend to focus more on its application in feature selection for essential proteins determination, which means that the swarm intelligence and machine learning are introduced to offer a systematic method for protein essentiality prediction. First, we gather some features potentially correlated with protein essentiality. Second, the optimal features are selected from our proposed Elite Search mechanism-based Flower Pollination Algorithm (ESFPA) method. Third, an ensemble classifier incorporated with our selected features is used to determine the protein essentiality, which is learned with Logistic model trees (LMT) [37], REPTree, a decision tree J48 implementing C4.5 algorithm [38], RandomTree, RandomForest [39,40], and a Naïve Bayes [41], as well as a Sequential minimal optimization (SMO) algorithm [42]. Finally, the performances of our method are compared in different aspects involving the comparison of ESFPA with the well-known feature selection methods like SFS, SBS, GA, ACO, PSO, and FPA, including the comparison of feature subset selected by ESFPA with other features and the comparison of our designed classifier with some existing classifiers. The experimental results show that our ESFPA method can achieve a better performance in terms of the 10-fold cross validation, which proves the effectiveness of our feature selection strategy and ensemble classifier for protein essentiality prediction. Simultaneously, ESFPA can offer an auspicious direction for predicting essential proteins based on swarm intelligence and machine learning.

The rest of this paper is organized as follows. In Section 2, the related work on the swarm intelligence algorithm used here is reviewed. In Section 3, the implementation of our feature selection strategy ESFPA and hybrid classifier is described in detail. The evaluation of the proposed method is discussed and analyzed in Section 4. Section 5 makes a summary of this study.

## 2. Preliminary

Flower Pollination Algorithm (FPA) [43] used in this study is a new type of swarm intelligence algorithm proposed in 2012, which considers the characteristics of flower pollination. Pollination can be achieved by cross-pollination or self-pollination. Cross-pollination may occur at long distance, and the pollinators can fly a long distance, thus it can be treated as global pollination, while self-pollination is regarded as local pollination. Global pollination and local pollination are considered as two main steps of FPA algorithm and are dominated by a switch probability *p* ∈ [0, 1].

In the global pollination, the pollinators carry the pollens a long distance because these pollinators, like insects, can move to a longer range, and pollination can occur from pollen of a flower of a different plant.

(1) $$x_{i}^{t+1}=x_{i}^{t}+L(x_{i}^{t}-G)$$

where $x_{i}^{t}$ is the pollen *i* at iteration *t*, and *G* is the current best solution that is found among all solutions at the current generation. The parameter *L* is a step size, i.e., the strength of the pollination. A Lévy flight as a type of random walk is used to represent that pollinators move over a long distance with various distance steps. Namely, *L* > 0 obeys the Lévy distribution,

(2) $$L\sim \frac{\lambda \Gamma (\lambda )\sin(\pi \lambda /2)}{\pi }\frac{1}{s^{1+\lambda }},(s\;>>\;s_{0}\;>\;0)$$

where Γ(λ) is a standard gamma function, and this distribution is valid for large steps *s* > 0.

Different from the global pollination, the local pollination does not require any pollinators and can occur from pollen of the same flower or different flowers of the same plant, and it can be denoted as

(3) $$x_{i}^{t+1}=x_{i}^{t}+\mu (x_{j}^{t}-x_{k}^{t})$$

where the pollen $x_{j}^{t}$ and $x_{k}^{t}$ are from the different flowers of the same plant species. Mathematically, if $x_{j}^{t}$ and $x_{k}^{t}$ are from the same plant species or select from the same population, this can be a local random walk if *μ* obeys a uniform distribution in [0, 1].

## 3. Materials and Methods

It is widely known that the prediction of essential proteins requires the use of selected features to judge the protein’s essentiality, that is, essential or nonessential. Most previous studies on feature selection investigated various topology and sequence features to assess how they related to essentiality. These features were ranked in the light of some criteria [15,44], and the higher sorting scores the features had, the more likely it is to be associated with essentiality. And given that it is difficult to decide whether the selected features have sufficient information, as many features as possible should be selected so as to ameliorate this problem. However, this will increase some ineffective and redundant features, which in turn will make machine learning face greater challenges.

To address these problems, we proposed a novel feature selection strategy, named ESFPA, to select the most suitable features by using swarm intelligence algorithm to find the optimal solution, which differs from those existing feature selection methods found in the previous literature. First of all, we collect 23 features closely related to the protein’s essentiality that consider not only from topological view but also from biological view. Topological features include DC, SC, EC, IC, Local Average Connectivity (LAC) [45], and Neighborhood Centrality (NC) [46], and biological features include protein module, Gene Ontology (GO), and subcellular location; at the same time the properties of protein domain, gene expression and orthology are also considered. Then, we know that the use of all the features does not necessarily results in the best performance. For this reason, an effective algorithm is required to select the most discriminative features to predict protein essentiality. Based on the aforementioned consideration, we apply a new kind of swarm intelligence algorithm named FPA with an elite search mechanism to feature selection, which is a critical step for protein essentiality prediction using the machine learning method. Finally, we build a hybrid classifier through the Vote method [47], which is a scheme for combining diverse classifiers to assemble a total of seven classifiers, i.e., Naïve Bayes, SMO, and five decision tree–based algorithms (J48, LMT, RandomForest, RandomTree, and REPTree).

**Figure 1:**
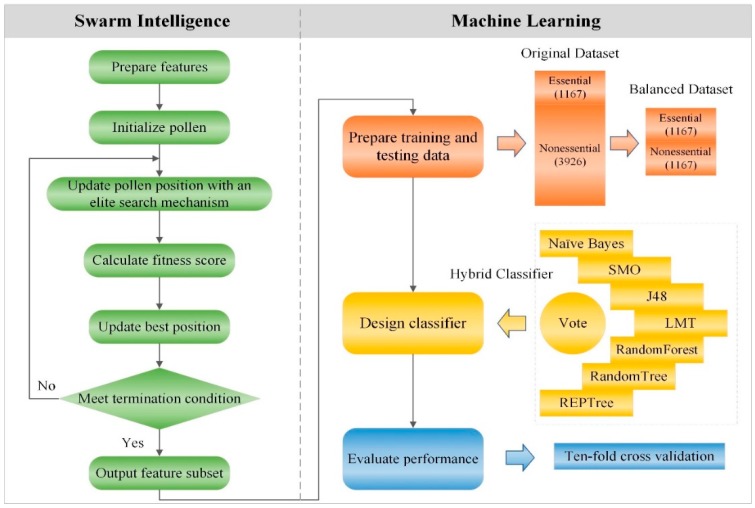
The system flowchart of Elite Search mechanism-based Flower Pollination Algorithm (ESFPA) feature selection strategy to predict protein essentiality.

Figure 1 displays a flowchart of our ESFPA method to predict protein essentiality using the hybrid classifier. As shown in Figure 1, ESFPA combines the swarm intelligence and machine learning. The former is used for feature selection and the primary steps contain preparing features, initializing pollen, updating pollen positions, calculating fitness scores and outputting feature subset, which will be presented in detail in Section 3.1, Section 3.2, Section 3.3, Section 3.4, Section 3.5 and Section 3.6. Based on the feature subset output by the former, the latter is to utilize our designed classifier to predict protein essentiality, which will be presented in detail in Section 3.7 and Section 3.8.

### 3.1. Data Source and Feature Preparation

The computational analysis is implemented based on the PPI network of *Saccharomyces cerevisiae* due to its data are the most complete in various species. The yeast PPI data used in this study was obtained from the DIP database [48] (version of 20101010). After removing the reduplicate interactions and self-connecting interactions, the data size was 5093, and the number of interactions was 24,743. The PPI network can be described as an unweighted and undirected graph *G* = (*V*, *E*) with the node set *V*, which represents the proteins, and the edge set *E*, which represents the interactions between two connected proteins. There are 1285 standard essential proteins are gathered from four databases: SGD [49], DEG [50], MIPS [51], and SGDP (http://sequence.stanford.edu/group/yeast_deletion_project).

To our knowledge, various types of features can uncover the protein essentiality at different levels. A broad variety of features are collected so that we can quantify their competence in distinguishing essential proteins from nonessential proteins. These features can be divided into topological and biological features. The detailed descriptions of each feature including their datasets are shown below.

#### 3.1.1. Topological Features

It is quite common to use network topology features in previous studies. For a given protein in the PPI network, DC is computed by the number of its neighbors, and a protein with the high degree in the PPI network tends to be essential [6,52]. SC is computed by the number of subgraphs that a protein participates in [11]. EC implies that a node can be considered central if it has a high eigenvector score [9]. IC is the harmonic mean lengths of paths ending at a protein [10]. LAC can measure the correlation between the protein and its neighbors [45]. NC focuses on the importance of the interactions that is closely associated with the protein essentiality [46].

#### 3.1.2. Biological Features

Gene Ontology (GO) [53] can depict the properties of proteins by a series of terms that are divided into three categories: biological process (BP), cellular component (CC), and molecular function (MF). The GO information is gained from the SGD database [49]. First, we calculate the functional similarity between any two proteins *s* and *t* by using formula GO(*s*,*t*) = GO(*s*) ∩ GO(*t*) according to the similarity of any two proteins in GO terms determines their functional similarity [54]. Then the GO score of each protein is defined as the sum of GO similarity of its neighbors.

Subcellular localization represents a specific location in cells in which a certain protein appears. We confirm that essential proteins are often located in certain subcellular locations [55,56] by examining the overall distribution of standard essential proteins and subcellular locations. The localization information is obtained from the COMPARTMENTS database [57]. Through our analysis, the proteins located in the nucleus are more likely to be essential, and this discovery is in agreement with the finding from Acencio and Lemke [17]. In addition to nucleus compartment, we also found that the essential proteins are mainly distributed in the cytosol, mitochondrion, endoplasmic reticulum, and cytoskeleton. If a protein presents in nucleus subcellular locations, its nucleus subcellular compartment score is represented as the proportion of this compartment to the total subcellular locations in which all standard essential proteins involved. The subcellular compartment scores of mitochondrion, cytoskeleton, golgi apparatus, peroxisome, cytosol, endosome, plasma membrane, extracellular space, vacuole, and endoplasmic reticulum are also defined in a similar way.

A protein module is a group of proteins that work together to perform some biological functions. We observe that essentiality is more likely to be the product of a module rather than a single protein [58], which is consistent with a recent study [14]. The module score of each protein is denoted as the number of protein modules acquired from CYC2008 [59] in which a protein is involved. Essential proteins tend to have higher module scores than nonessential proteins.

Some synthetical biological features are also used here. The orthologous property in the ION [12] is utilized because essential proteins are more evolutionarily conserved than nonessential proteins [15]. We also employ two measures, PeC [60] and WDC [61], to demonstrate the impact of the gene expressions on essentiality. Protein domains are the function and structure units of proteins, and it is well known that the proteins with more domain types that rarely exist in other proteins are more likely to be essential [62]. Based on this observation, we know that protein domain is useful for the prediction of protein essentiality, and we take advantage of domain information in UDoNC [62].

Eventually, a collection of 23 features related to the essentiality is obtained, and then 18 features are remained by preprocessing to remove those less predictive subcellular compartment according to the calculation of subcellular compartment score for each compartment.

### 3.2. Golden Standard of Features

The Pearson correlation coefficient (PCC) can assess the correlation between different features. We use *cc_ij_* to measure whether the features are distinct which is used to rank the features and the correlation value between two features *i* and *j* are shown as the following equation.

(4) $$cc_{ij}=\frac{\sum_{m=1}^{n}\left(x_{i}(m)-{\bar{x}}_{i})(x_{j}(m)-{\bar{x}}_{j}\right)}{\sqrt{\sum_{m=1}^{n}{\left(x_{i}(m)-{\bar{x}}_{i}\right)}^{2}}\sqrt{\sum_{m=1}^{n}{\left(x_{j}(m)-{\bar{x}}_{j}\right)}^{2}}}$$

Then, the correlation value of feature *i* is computed as the following equation.

(5) $$cv_{i}=\frac{\sum_{j=1}^{fn}\left|cc_{ij}\right|\;}{fn-1}\;(i\neq j)$$

where *fn* is the number of all the features. The features with the lower values of *cv_i_* are the more distinguishable and informative. We sort these features in ascending order according to their corresponding correlation values, and the features of the ranking in top two-thirds are more distinguishable, which is considered as the gold standard.

### 3.3. Encoding and Initializing

In this subsection, we gather 18 features processed in the first step to encode a pollen. The positions of the pollen are converted into the binary form, which are denoted by a string of bits. Each bit denotes the absence or presence of a feature, namely, each bit is labeled as 1 if a feature is selected to consist of the new feature subset and 0 otherwise. We will give a simple example in order to better understand the encoding operation. For example, if fea_1_, fea_2_, fea_3_, fea_4_, and fea_5_ are original features and the position of a pollen is 1, 1, 0, 1, and 0, respectively, it means that fea_1_, fea_2_, and fea_4_ are selected as ultimate feature subset.

The population of pollen is initialized, and each pollen is given a random position with the *D*-dimensional space, where *D* stands for the number of features.

### 3.4. Fitness Score

The linear combination of feature performance and the number of features is represented as a fitness score that is measured by the superiority of features. The prediction of features is either TD (true distinct), FD (false distinct), TI (true indistinct), or FI (false indistinct). TD denotes that true distinct features that belong to the gold standard are correctly identified as distinct, FD denotes that indistinct features that do not belong to the gold standard are incorrectly identified as distinct, TI denotes that indistinct features that do not belong to the gold standard are correctly identified as indistinct, and FI indicates that distinct features that belong to the gold standard are incorrectly identified as indistinct. The accuracy of selected features is defined as follows:

(6) $$Fperformance=\frac{TD+TI}{TD+TI+FD+FI}$$

In order to select the most representative features subset, we use the fitness score, which is denoted as follows:

(7) $$Fitness\;Score=\alpha \times Fperformance+(1-\alpha )\times \frac{1}{\left|N\right|}$$

where |*N*| is the number of features, and parameter *α* is a constant between zero and one that measures the relative importance. It implies that the higher the fitness score of the feature subset, the more likely it is to make a greater contribution to predicting protein essentiality. Parameter *α* is given a larger value that is set to 0.8 because the importance of the first part is greater than the second part in the above formula.

### 3.5. Update Rule with an Elite Search Mechanism

The key to the task is to select the appropriate features and the update operation of this algorithm can achieve this goal. To construct a binary vector, the following equations are added to the standard FPA [35].

(8) $$S(x_{i}^{j}(t))=\frac{1}{1+e^{x_{i}^{j}(t)}},$$

(9) $$x_{i}^{j}(t)=\left\{ \begin{array}{ll} 1 & \;if\;S(x_{i}^{j}(t))>\delta \\ 0 & \;otherwise \end{array}\right.$$

where δ satisfies a uniform distribution in [0, 1].

In the update process, an elite search mechanism is applied to improve the performance of the FPA algorithm here. The main idea of this elite search mechanism is to make the pollen individual continuously fly to the global optimal solution so that the convergence of FPA algorithm can be accelerated, and its operation is specified as the following equation:

(10) $$x_{i}^{j}(t)=\left\{ \begin{array}{ll} cons\times G* & \;if\;x_{i}^{j}(t)-G*\neq 0\\ x_{i}^{j}(t) & \;otherwise \end{array}\right.$$

where G* is the global optimal pollen, i.e., the global optimal solution. The parameter *cons* is a constant between zero and one, and its value is set as two-thirds in this study. It should be noted that we only focus on the top two-thirds of each pollen in this equation here. This equation indicates that the first two-thirds of bits of the current pollen minus those of the global optimal pollen and the bits that do not equal to zero are replaced by the corresponding global optimal pollen. Such an operation can ensure that the current pollen flies toward the global optimal pollen.

### 3.6. Outputting Feature Subset

The feature selection is the process of picking the most informative features so that the selected feature subset is more adaptive for classification task. The global optimal pollen is extracted by our FPA algorithm with the elite search mechanism that contains more effective features among all the pollen individuals, which can be the most suitable choice of the feature subset. In the final feature subset, thirteen features are decided to be the most predictive and representative and these features contain DC, EC, SC, PeC, ION, module, UDoNC, nucleus, cytosol, mitochondrion, endoplasmic reticulum, cytoskeleton, and golgi apparatus.

### 3.7. Preparing Training and Testing Data

Proteins in a PPI network can fall into three categories: essential proteins, nonessential proteins, and essentiality unknown proteins. We only consider the first two categories, namely, essential and nonessential, because the third category cannot provide any information [63]. There are 1285 standard essential proteins in total, while 1167 proteins exist in the PPI network. In our study, 1167 proteins are treated as the essential proteins, and the remaining 3926 (=5093 − 1167) proteins are regarded as the nonessential proteins, in so-called imbalanced datasets. It should be admitted that the imbalanced datasets affect the performance of machine learning technologies [64]. Therefore, we use 1167 essential proteins and randomly select 1167 from 3926 nonessential proteins to compose the balanced dataset. This process is executed 10 times to achieve 10 different balanced datasets.

### 3.8. Classifier Design and Evaluation

The prediction of protein essentiality belongs to a binary classification problem [65]. To measure the predictive capability of our ESFPA method, the classification of essential proteins is performed using the WEKA software package [66] to create an ensemble classifier which is the integration of seven different learning algorithms. These seven algorithms include five decision-tree based algorithms (LMT, REPTree, J48, RandomTree, RandomForest) and a Naïve Bayes, as well as an SMO algorithm, and they have diverse but complementary performances.

We use the WEKA software package [66] to perform 10-fold cross validation to make an evaluation for our ESFPA method, the dataset is separated into 10 equal parts, nine folds are used to train the classifiers, and the remaining one fold is used for testing [55]. For each test, the classification and evaluation are carried out in 10 runs, and the experimental result is taken from the average of these 10 runs.

For a given protein, there are 13 more informative features selected from ESFPA as input variables for our designed classifier that can predict protein essentiality with good performance. The inputs of the classifiers include the features of each protein and the corresponding class labels. Note that all experiments in this study are executed in the WEKA package.

## 4. Results and Discussion

### 4.1. Comparison of All the Balanced Datasets

**Figure 2:**
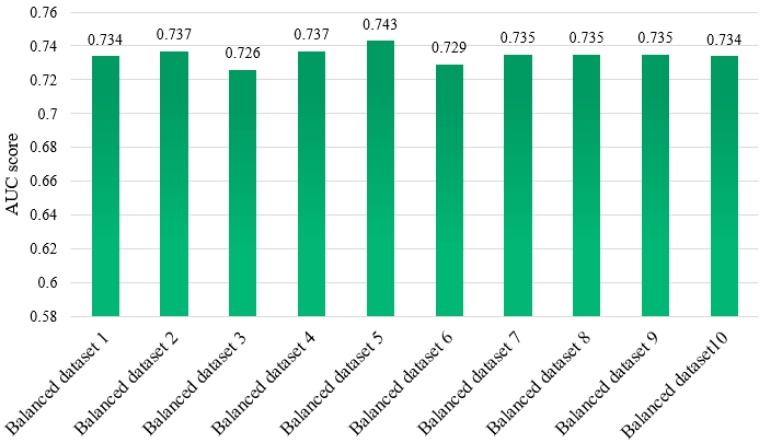
Area under the receiver operating characteristic (ROC) curve (AUC) scores for our classifier trained on 10 balanced datasets.

To analyze the performance of all balanced datasets before formally predicting protein essentiality, we use 1167 essential proteins and then randomly select 1167 from 3926 nonessential proteins to compose the balanced dataset. This process is executed 10 times to obtain 10 different balanced datasets, and the difference between these datasets is that the subset of nonessential proteins in each dataset are not the same. Our hybrid classifier is trained in each of the 10 balanced datasets, and their corresponding performance is assessed in terms of the area under the receiver operating characteristic (ROC) curve (AUC) scores, which is widely used to assess the performance for computational methods, as shown in Figure 2. We can see that there is no significant difference in the prediction performance of these ten balanced datasets according to the AUC scores yielded by 10-fold cross validation in the WEKA software. Thus, we randomly select one of the ten balanced datasets to continue the following experimental analysis on the balanced dataset.

### 4.2. Comparison of the Balanced Dataset with Imbalanced Dataset

**Figure 3:**
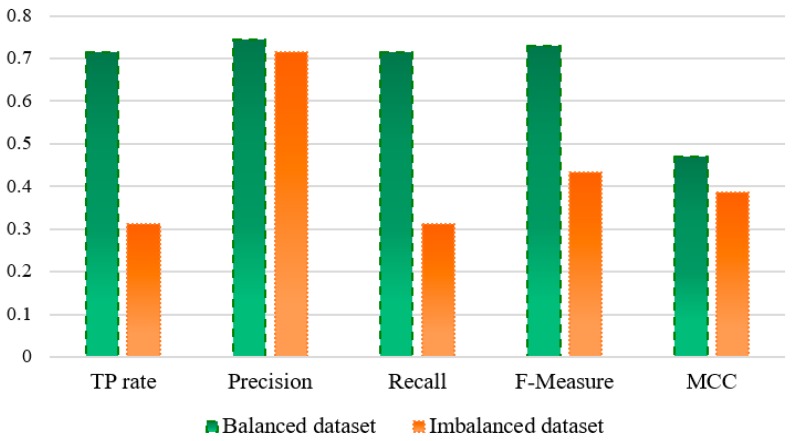
Comparison of the balanced dataset with imbalanced dataset.

From the previous work, we have known that the imbalanced datasets affect the performance of machine learning technologies [64]. To verify this finding, we randomly selected one of the 10 balanced datasets to perform this experiment because there is no difference in their prediction performance from the previous analysis. Then, we compared the performance between the balanced dataset and imbalanced dataset in the prediction of protein essentiality (Figure 3) by using True Positive rate (TP rate), Precision, Recall, F-Measure, and Matthews Correlation Coefficient (MCC) in WEKA software. As shown in Figure 3, we can see that the performance of the balanced dataset is better than those of the imbalanced dataset. Particularly, compared with the imbalanced dataset, the balanced dataset performs better with more than two times improvement in terms of Recall (balanced dataset: 0.715, imbalanced dataset: 0.311), which further confirms the correctness of using a balanced dataset in our study.

### 4.3. Comparison of Different Feature Selection Methods

**Figure 4:**
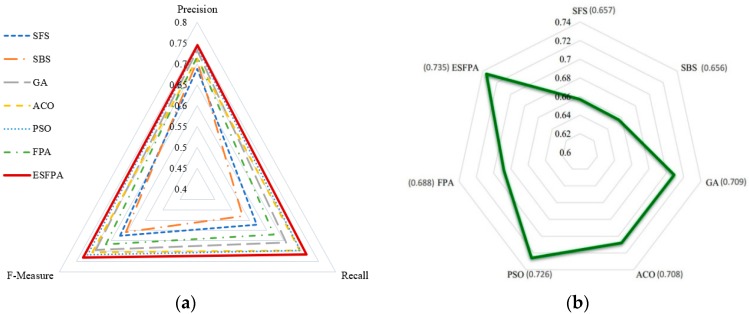
Comparison of our ESFPA method and some well-known feature selection methods. (**a**) shows the comparison results in terms of Precision, Recall, and F-Measure; (**b**) shows the comparison results in terms of AUC scores.

We analyze the ability of different feature selection methods for the prediction of protein essentiality and demonstrate them by using some evaluation indexes including Precision, Recall, F-Measure values, and AUC scores (Figure 4a,b). The specific details of Precision, Recall, and F-Measure are recorded in Table 1 and note that all feature selection methods are implemented on the balanced dataset. A Precision of 0.689, Recall of 0.571, F-Measure of 0.624, and AUC score of 0.657 are obtained for the SFS method. At the same time, a Precision of 0.708, Recall of 0.530, F-Measure of 0.607, and AUC score of 0.656 are obtained for the SBS method. The performance of SFS and SBS is worse than that of all the swarm intelligence algorithms in feature selection (Figure 4, Table 1). For Precision, Recall, F-Measure, and AUC scores, all four values of ESFPA are higher than those of other feature selection methods, which indicates that its performance outperforms those obtained by some state-of-the-art feature selection methods presented here. It can be observed that our ESFPA algorithm significantly improves the performance of the FPA algorithm without incorporating an elite search strategy we designed, which also proves that our improved ESFPA algorithm is quite effective compare to the original FPA algorithm for feature selection to predict protein essentiality. The performance of various feature selection methods on imbalanced dataset is also reported in Table 2 to show the differences between them. Comparing Table 2 with Table 1, we can clearly see that no matter which methods, the results on the imbalanced dataset are worse than those on the balanced dataset, which further illustrates that the imbalanced dataset has a great impact on the performance of various methods.

### 4.4. Comparison of Different Feature Subsets

**Figure 5:**
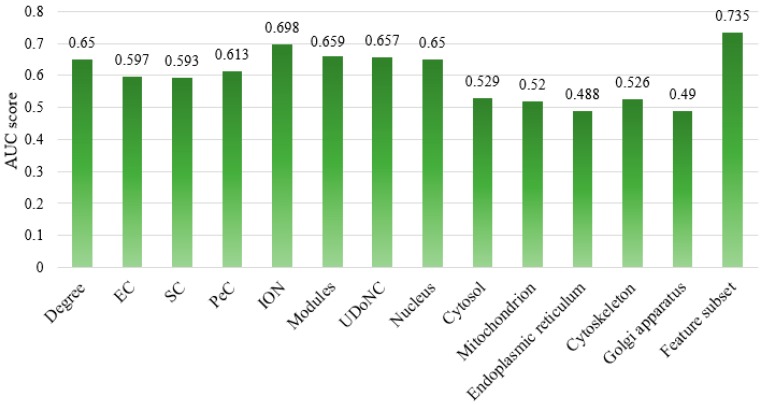
AUC scores of our selected features and those using individual features.

In order to further estimate the performance of the integrated features selected from our ESFPA algorithm, we train the hybrid classifier in the balanced dataset with our selected features and with individual features, respectively. The AUC scores shown in Figure 5 indicate that our feature subset is 0.735, which is clearly higher than any individual features and confirm that the selected features from our ESFPA algorithm has the strongest predictive power. Also, the values of Precision, Recall, and F-Measure for each feature subset are listed in Table 3, and it shows that the performance of the feature subset selected from our ESFPA algorithm is superior to those that consider only individual features. Our selected features display a great improvement on the AUC score and F-Measure in comparison with other single features. It is revealed that the rational feature selection based on our ESFPA algorithm can obtain better feature subset with higher F-Measure and AUC score, which suggests that our proposed feature selection strategy ESFPA can find the most predictive features taking into account both the topological and biological information to predict protein essentiality.

### 4.5. Comparison of Diverse Classifiers Performance

To understand the contribution of each feature added to the classifier, we chose to perform in-depth analysis of the features that have a greater impact on the results. Meanwhile, the predictive power of our hybrid classifier was also assessed. The WEKA package was used to train and evaluate the classifiers to classify whether a protein is essential. We compared our new classifier (NC) that is chosen as the predictor to recognize protein essentiality with diverse classifiers: Naïve Bayes (NB), Sequential minimal optimization (SMO), J48, Logistic model trees (LMT), Random Forest (RF), RandomTree (RT), and REPTree (REPT). All parameter settings for each classifier are carefully provided in Supplementary Materials, Table S1. The comparison results in terms of Precision (Pre), Recall (Rec), and F-Measure (F) are shown in Table 4. For feature Degree, EC, SC, PeC, and UDoNC, the performance of NB and SMO is worse than that of other classifiers. F-Measure values of J48, LMT, and NC are the best for Degree. The F-Measure values of features ION, modules, and nucleus are better for NB. For SMO, in addition to these three features, our feature subset also performs well. The F-Measure values of ION, Nucleus and our feature subset are better for J48 and LMT. For NC, the F-Measure values of features Degree, ION, and nucleus are better, which suggest that these features make the greater contribution. And our feature subset gets the highest F-Measure for NC. For our selected feature subset, the one that reflects the better performance is our new classifier integrating seven algorithms (Naïve Bayes, SMO, J48, LMT, RandomForest, RandomTree, and REPTree) by using the Vote method performed by the WEKA package. Unlike those single classifiers, we consider that diverse classifiers are complementary and have their own advantages. We combine the advantages of multiple classifiers to design new classifier and therefore the better result can be obtained. Through our experimental analysis, the combination of our selected feature subset and designed new classifier can achieve better performance.

## 5. Conclusions

Essential proteins are necessary for the support of cell life. There is no doubt that the prediction of protein essentiality is of great significance in guiding the research to discovering disease genes and drug targets. In our study, we first collected a large number of features related to essentiality, including both topology-based features, such as DC, SC, EC, IC, LAC, and NC, and biology-based features, such as GO, subcellular location, module information, protein domain, gene expression, and orthology. Then, the FPA algorithm with an elite search mechanism named ESFPA was used to select suitable feature subset, which are more potent in reflecting the essentiality of proteins. After that, an ensemble classifier, the one that including seven different learning algorithms, was used to distinguish whether a protein is essential. Finally, the performance of our ESFPA method was tested by a series of experiments, which demonstrated the competitiveness and effectiveness of our ESFPA method for predicting protein essentiality. The results indicated that our hybrid classifier combined with diverse classifiers worked better than other existing classifiers, and the most predictive features selected from our ESFPA method resulted in better prediction performance. In summary, our method presented in this paper may shed some light on the researches of protein essentiality in swarm intelligence and machine learning. It is believed that with the availability of more data, we can make it convenient to use more topological and biological information to further predict protein essentiality.

## Figures and Tables

**Table 1:** Comparing the performance of different feature selection methods on balanced dataset.

Feature Selection Methods	Evaluation Criterion		
	Precision	Recall	F-Measure
SFS	0.689	0.571	0.624
SBS	0.708	0.530	0.607
GA	0.733	0.657	0.693
ACO	0.713	0.696	0.704
PSO	0.740	0.697	0.718
FPA	0.718	0.620	0.665
**ESFPA**	**0.745**	**0.715**	**0.730**

**Table 2:** Comparing the performance of different feature selection methods on imbalanced dataset.

Feature Selection Methods	Evaluation Criterion		
	Precision	Recall	F-Measure
SFS	0.554	0.158	0.245
SBS	0.544	0.134	0.215
GA	0.654	0.275	0.387
ACO	0.693	0.269	0.388
PSO	0.708	0.314	0.436
FPA	0.691	0.282	0.400
**ESFPA**	**0.716**	**0.311**	**0.434**

**Table 3:** Comparing the performance evaluations of different feature subsets.

Feature Subsets	Evaluation Criterion		
	Precision	Recall	F-Measure
Degree	0.667	0.598	0.631
EC	0.660	0.401	0.499
SC	0.665	0.376	0.481
PeC	0.747	0.341	0.468
ION	0.717	0.655	0.684
Modules	0.715	0.530	0.609
UDoNC	0.759	0.459	0.572
Nucleus	0.654	0.638	0.646
Cytosol	0.594	0.181	0.277
Mitochondrion	0.512	0.848	0.639
Endoplasmic reticulum	0.484	0.352	0.408
Cytoskeleton	0.736	0.081	0.147
Golgi apparatus	0.492	0.669	0.567
**ESFPA-feature subset**	**0.745**	**0.715**	**0.730**

**Table 4:** Comparing the performance of diverse classifiers.

Feature	Classifier	Pre	Rec	F	Feature	Classifier	Pre	Rec	F
Degree	NB	0.714	0.227	0.345	EC	NB	0.719	0.162	0.264
	SMO	0.686	0.150	0.246		SMO	0.736	0.127	0.216
	J48	0.667	0.598	0.631		J48	0.653	0.509	0.572
	LMT	0.667	0.598	0.631		LMT	0.634	0.546	0.587
	RF	0.661	0.576	0.615		RF	0.533	0.522	0.528
	RT	0.664	0.586	0.623		RT	0.531	0.524	0.528
	REPT	0.658	0.599	0.627		REPT	0.629	0.543	0.583
	NC	0.667	0.598	0.631		NC	0.660	0.401	0.499
SC	NB	0.670	0.054	0.100	PeC	NB	0.810	0.244	0.375
	SMO	0.714	0.017	0.033		SMO	0.846	0.175	0.290
	J48	0.655	0.513	0.575		J48	0.759	0.295	0.425
	LMT	0.638	0.542	0.586		LMT	0.682	0.402	0.506
	RF	0.532	0.530	0.531		RF	0.542	0.538	0.540
	RT	0.529	0.532	0.531		RT	0.541	0.538	0.540
	REPT	0.618	0.559	0.587		REPT	0.660	0.402	0.499
	NC	0.665	0.376	0.481		NC	0.747	0.341	0.468
ION	NB	0.722	0.638	0.678	Modules	NB	0.715	0.530	0.609
	SMO	0.715	0.663	0.688		SMO	0.715	0.530	0.609
	J48	0.711	0.626	0.665		J48	0.715	0.530	0.609
	LMT	0.707	0.679	0.692		LMT	0.715	0.530	0.609
	RF	0.662	0.568	0.611		RF	0.715	0.530	0.609
	RT	0.659	0.584	0.619		RT	0.715	0.530	0.609
	REPT	0.708	0.638	0.671		REPT	0.715	0.530	0.609
	NC	0.717	0.655	0.684		NC	0.715	0.530	0.609
Nucleus	NB	0.654	0.638	0.646	UDoNC	NB	0.828	0.226	0.355
	SMO	0.654	0.638	0.646		SMO	0.902	0.039	0.076
	J48	0.654	0.638	0.646		J48	0.722	0.544	0.620
	LMT	0.654	0.638	0.646		LMT	0.716	0.553	0.624
	RF	0.654	0.638	0.646		RF	0.693	0.693	0.577
	RT	0.654	0.638	0.646		RT	0.689	0.495	0.576
	REPT	0.654	0.638	0.646		REPT	0.690	0.589	0.636
	NC	0.654	0.638	0.646		NC	0.759	0.459	0.572
Feature subset	NB	0.807	0.380	0.516					
	SMO	0.740	0.710	0.725					
	J48	0.728	0.717	0.722					
	LMT	0.736	0.723	0.729					
	RF	0.710	0.729	0.720					
	RT	0.644	0.661	0.652					
	REPT	0.705	0.689	0.697					
	NC	0.745	0.715	0.730					

## Supplementary Materials

The following are available online. Table S1: The parameter settings of classifiers contain Naïve Bayes, Sequential minimal optimization (SMO), J48, Logistic model trees (LMT), Random Forest, RandomTree, and REPTree. All parameters are based on the WEKA software package. Note that the data and code can be download from here: https://github.com/mfbox/ESFPA.
