# Supplementary material for: Feature Selection via Swarm Intelligence for Determining Protein Essentiality
Source: Molecules. 2018 Jun 28;23(7):1569. doi: 10.3390/molecules23071569 (PMC6100311; doi:10.3390/molecules23071569)
Supplement: Supplementary file 1 [file molecules-23-01569-s001.zip › molecules-314677-supplementary.pdf]

Appendix A

Table S1. The parameter settings of classifiers contain Naïve Bayes, Sequential minimal optimization (SMO), J48, Logistic model trees (LMT), Random Forest, RandomTree and REPTree. All parameters are based on the WEKA software package.

Classifiers	Parameter Setting
Naïve Bayes	debug: False
	useKernelEstimator: False
SMO	Calibrator: Logistic
	debug: False
	kernel: PolyKernel
	numFolds: -1
	randomSeed: 1
J48	binarySplits: False
	confidenceFactor: 0.25
	debug: False
	minNumObj: 2
	numFolds: 3
	reducedErrorPruning: False
	saveInstanceData: False
	seed: 1
	unpruned: False
	useLaplace: False
LMT	convertNominal: False
	debug: False
	fastRegression: True
	minNumInstances: 15
	numBoostingIterations: -1
	splitOnResiduals: False
	useAIC: False
	weightTrimBeta: 0.0
RandomForest	debug: False
	maxDepth: 0
	numFeatures: 0
	seed: 1
RandomTree	KValue: 0
	debug: False
	maxDepth: 0
	minNum: 1.0
	seed: 1

REPTree	debug: False<br>maxDepth: -1<br>minNum:2.0<br>minVarianceProp: 0.001<br>noPrunning: False<br>numFolds: 3<br>seed: 1
